# Insects as Carriers of Tropical Diseases

**Published:** 1910-12-03

**Authors:** G. C. Low

**Affiliations:** Lecturer on Tropical Diseases, Post-Graduate College, West London Hospital.


### Hospital Clinics.

### INSECTS AS CARRIERS OF TROPICAL DISEASES.

By G. C. LOW, M.D., Lecturer on Tropical Diseases, Post-Graduate College, West London Hospital.

**Delivered at the West London Hospital, Hammersmith, W., on Tuesday, October 25, 1910.**

Gentlemen,—I propose this afternoon to lecture to you upon the subject of the part played by insects in the spread of tropical diseases. The part which they play is a very great one, and it is no exaggeration to say that most of the important tropical diseases owe their existence to, or part of their stage has to be passed in, some insect as an intermediary. The vast bulk of diseases in this country owe no such relationship to insects, though perhaps some are more commonly spread by means of the direct transference of their germs by flies than is generally supposed. If we take the simplest instance of an insect transmitting a disease we may choose the case of typhoid fever or cholera and the part which flies play in the spread of those diseases. This part is a very real one. During the war in South Africa the large epidemics of typhoid were, without doubt, to a great extent produced by flies. How this should be is easy to see. For example, flies settle on typhoid stools in trenches or faulty latrines; then they go into the adjoining tents or buildings, and next settle on the milk or other food. Milk being a good cultural medium for the bacilli, the latter multiply there; and then when the milk is drunk later the individual ingests a powerful culture of the germs, and in all probability in due course goes down with typhoid fever.

### Flies that Carry Cholera.

It is the same with cholera; in that instance Koch's comma bacillus is transferred to the food by flies. Typhoid, cholera, and quite likely bacillary dysentery as well, all may be spread then by the direct transference of their specific germs from the fæces by the feet of flies to the food of man, and thus you will at once see the importance of preventing the access of flies to food and into houses generally. Anthrax is another disease where flies have been known to help in its spread. There is one instance at least on record where this actually happened. A man in a field crushed a big bluebottle on the back of his hand, and where he crushed it a malignant pustule afterwards developed. Subsequent investigation showed that there had been a dead cow in the next field which had died of anthrax, and the fly evidently had been on this carcase, had got anthrax bacilli on its legs, had flown from there on to the back of the man's hand, and when it was crushed some of the bacilli had got into one of the hair follicles and so set up the malignant pustule. These are examples of the feet and legs of flies acting in much the same way as the inoculating-needle in the laboratory—taking a germ from one place and transplanting it to another. Another disease which has recently been shown to be spread by insects—by fleas—is plague. This is not quite so simple as the cases just mentioned, as the plague bacillus apparently passes through the stomach and intestine of the flea first. During its passage it does not undergo any special development, however, so it may be more or less classified in the category of a direct transference of a bacterial germ from one host to another. Plague is a disease of rats, and in those animals it usually takes the form of a septicæmia. The flea bites the septicæmic rat and ingests the plague bacilli; these passing into its stomach, then into its intestines, and so eventually are voided with the fæces. As the rat dies its fleas may pass on to the skin of man, and these on defæcating will expel plague bacilli, which either enter by the hair follicles or through the puncture wounds made by the same or other fleas, and so bring about the infection. Most people who go with bare legs and feet get bitten on these parts, and the bubo comes as a result in the groin (bubonic plague).

The pneumonic form of plague has probably nothing to do with the flea. The germs coughed out by the invalid are inhaled by others, and so the disease is propagated. These cases are very dangerous to go near, and intimate contact with such should always be avoided. The bubonic cases are not so important, and provided the rats about the place are not infected there should not be further cases.

### How Plague is Brought.

Cases of plague come from time to time to the Thames in steamers from India, and some of these have found their way into the hospitals at the docks without anything untoward happening. For plague to get into a place and take hold rats must be present, and rat plague start first. The pneumonic type I have just alluded to is probably an exception to this. There is the well-known instance which occurred in Vienna, where a laboratory assistant who infected himself in a laboratory was taken into a ward for pneumonia—in reality plague pneumonia—and the nurses and doctors who attended the case all got pneumonic plague and died from it.

We may pass now to a higher group of organisms than these we have been discussing; namely, to the protozoa, the lowest forms of animal life. Up till quite recently we believed that one of these—namely, the trypanosome—was spread directly from animal to animal by means of the proboscis of the tsetse-fly as if by an inoculating-needle, but now it is probable that a proper development of this parasite has to take place within the stomachs of such insects.

### The Agency of Flies.

Experimentally, of course, direct transference of such a parasite from rat to rat, say, is very easy; but from what has recently been found out, such a mechanical transference of the trypanosome by the proboscis of the tsetse-fly in Nature is probably of fairly rare occurrence. Two years or so ago Kleine in Africa showed by experiments that the trypanosome develops for a varying period of time in the interior of the fly, and that it is only after later dates that such a fly becomes infective. From quite a short time after biting (a few hours) to this later date the flies remain non-infective, but in interrupted feeding experiments there is still a chance of the transference of the parasite directly; *i.e.* if a fly is feeding on a heavily infected animal and is disturbed, and immediately settles on and bites another, it might transfer the parasite. In the case of some of the other protozoal parasites, and in the case of others higher in the animal kingdom still, a proper development or metamorphosis has to take place in some of the tissues of the insect, this resulting in the production of forms of the parasite which are transferred to the next host. Some of these life histories are very complicated and not so easy to understand. First I shall call your attention to malaria, which is perhaps the best known of these, and then afterwards you will better understand some of the other diseases which I shall describe later.

### The Life History of the Malarial Parasite.

There are two phases of the malarial parasite: first, the intracorporeal or asexual phase in man; second, the extra-corporeal or sexual stage in the mosquito. In the early stages in man's blood the malarial parasites are small protozoal organisms which inhabit the red blood corpuscles; these grow larger, ultimately filling the whole or nearly the whole of the corpuscle; then the black pigment collects in a mass in the centre, the protoplasm breaking up around this into a number of spores. The corpuscle next ruptures, the spores escaping into the plasma, and these in turn penetrate and enter new red blood corpuscles, and so start the cycle again.

When this sporulation, as it is called, is going on, however, we see other bodies which do not behave in a similar manner; these are the gametes or sexual forms, which when sucked out of the blood by suitable mosquitoes will develop and go through the sexual phase.

### The Mosquito.

When the mosquito sucks up ordinary malarial blood it will, of course, take up all the forms; but the asexual parasites, rings, half-grown and sporulating shapes, are simply digested and disappear. With the others, however, the gametes, complicated changes now take place. The crescents, the gametes of malignant malaria, become spherical, the gametes of the benign fever being spherical from the first. Some of these bodies are males, other females; and eventually from the former actively moving flagella are given off. These are similar in nature to the spermatozoa of higher animals. A single flagellum penetrates one of the female cells, and sexual union or zygosis is thus accomplished. The resulting body elongates out, and is now known as a travelling vermicule. The function of this body is to penetrate the walls of the mosquito's stomach, which consist of the following four layers: (1) A delicate cuticula, (2) a layer of cubical cells, (3) an elastic lamina, and (4) a muscular coat of circular and longitudinal fibres. Having accomplished this the vermicule comes to rest in the outer part of the stomach wall, where it again becomes circular and is then known as the oocyst. This latter body grows and grows until eventually it protrudes from the stomach of the mosquito like a little wart or hernia. At the same time as this goes on minute changes are taking place within its interior. We get a segmentation of the protoplasm into what are known as blastophores, and later the appearance of spinous bodies on the walls of these; still later the blastophores disappear, and the cavity now becomes packed with these small spinous bodies, which are technically known as sporozoits. Now the wall of the cyst ruptures, the sporozoits being set free into the tissues of the mosquito, their ultimate fate being to be collected and stored up in the salivary glands, organs situated in the anterior part of the thorax of the insect. From the salivary glands the salivary duct leads to the proboscis, and when the infected mosquito next bites man sporozoits *plus* the saliva will be injected into the wound. The little organisms soon find their way to the red corpuscles, penetrate their walls and enter into their substance, become circular, and then from these, the young rings, the asexual or intracorporeal cycle again starts and the chain is complete.

These changes just described take a definite time to accomplish; at a suitable temperature they take ten days or a little longer. After one is bitten by the infected mosquito there is an incubation period of ten to twenty days, and then the fever appears. All mosquitoes will not act as suitable intermediaries for the malarial parasite; only certain ones of the sub-family of the anophelinæ are suitable. Malaria, then, is an example of a disease where the specific parasite has to undergo a development in an intermediate host, in this case a suitable anopheline. If such a development did not take place the disease would come to an end, because when a person who had malaria died he would not transmit it to anyone else, his parasites dying with him.

Further, if one could cut the chain of the cycle by removing all mosquitoes, *i.e.* the intermediate hosts, the disease would again come to an end. But this will be better dealt with in a future lecture on the prevention of tropical diseases.

### Yellow Fever.

I will next speak of a disease the germs of which have again to pass through a mosquito, and which in many ways has a close analogy to malaria—namely, yellow fever. Strangely enough, though we know the disease is spread by mosquitoes, we do not yet know the parasite either in man or the mosquito. What we know of the ætiology of the disease is due to the work of the Americans in Havana, who, working on the analogy that the malady might be similar to malaria, discovered that a special mosquito, the *Stegomyia calopus*, is the transmitting agent. They fed such mosquitoes on yellow-fever patients, and then allowed these insects to bite susceptible individuals at varying periods of time afterwards, eventually finding that if puncture took place after the thirteenth day the disease was acquired. It had been noticed that people could be put into beds in which yellow-fever cases had died, and where even there was black vomit, and yet they did not get yellow fever; whereas, on the other hand, when placed in specially constructed rooms with infected mosquitoes they invariably got it. To summarise; our knowledge of the part the mosquito plays in yellow fever is as follows. We know that in the blood of yellow-fever cases on the second, third, and even in some instances on the fourth day there is some kind of infective agent circulating in the peripheral blood. Mosquitoes of the genus *Stegomyia* and of the species *calopus* when they suck up such blood become infected, and after a period of thirteen days or so become infectious. Susceptible individuals bitten by such infected mosquitoes readily acquire the disease, the incubation period being a short one of only two or three days. The life of a *Stegomyia* may be as long as sixty days, and once infected they appear always to remain so; therefore the number of people they may bite and infect in such a time may be very great. Here again, if we can cut the mosquito out, I mean destroy or greatly diminish all the *Stegomyia* in a place, yellow fever must come to an end; and this, as I shall tell you in my next lecture, has actually been done in Havana and other parts of America where yellow fever used to be rife. You can sit with a yellow-fever case all day, and not run the slightest risk of acquiring the disease, provided no mosquitoes (*Stegomyia calopus*) are present; and so also, if by means of mosquito nets this mosquito is kept out, there is no danger. The interesting feature of the disease is that the parasite has never been seen. Infected blood passed through a porcelain filter is still said to retain its infectivity; therefore the parasite, whatever it may be, is said to be ultramicroscopical.

### Dengue.

I can give you another example; namely, dengue. It has been shown in the last few years that this troublesome disease is also spread by mosquitoes; namely, by the ordinary brown domestic mosquito, *Culex fatigans*. No one has demonstrated a parasite in the blood of dengue cases so far; but if the mosquito mentioned sucks the blood of such cases it becomes infected, and in time infectious, giving dengue to susceptible people whom it may bite.

There is yet another disease—namely, horse sickness of South Africa—that is caused by another of these ultramicroscopical parasites, and which is also very likely spread by mosquitoes.

We now pass to another disease, *filariasis*, a very interesting and important tropical ailment, and one caused by a nematode worm, the *Filaria bancrofti*. The worms, of course, are comparatively high up in the animal kingdom—very much higher, for example, than the lowly protozoa we have been chiefly dealing with so far. Still, though this is so, this individual worm, the *Filaria bancrofti*, has to pass through a metamorphosis in mosquitoes in a somewhat similar manner to the parasites just described. The adult forms of *Filaria bancrofti* inhabit the lymphatics of man; they are quite visible to the naked eye, measuring as much as 2 or even 3 inches in length, and resembling fine pieces of catgut or thread; they are bisexual, and the females give birth to microscopic embryos which circulate in the blood, appearing in the peripheral blood by night and disappearing during the day. These embryos are shaped like little snakes or eels, and measure 1/90th of an inch in length (.317mm.) by 7.5 $\mu$ in breadth; *e.g.* the breadth of a red blood corpuscle. At night, as I have said, the embryos appear in the peripheral blood; so that if you go into a hospital in the tropics, and bleed the natives between 10 P.M. and midnight, you will find in a certain number of them these embryo filariæ. During the day they disappear, going into the heart, the lungs, and large vessels of the thorax, where they remain till evening comes round again. How they do this is quite unknown.

### The Filaria.

All insects that bite a filariated individual must necessarily suck up the embryos into its stomach; but in the majority of instances—fleas, bugs, lice, etc.—they (the embryos) are digested and eventually disappear. If, on the other hand, *Culex fatigans* or some of the anophelines come along quite a different thing happens. The embryos erupt from their sheaths, penetrate the wall of the stomach, bore their way into the thoracic muscles, and there come to rest. After this they soon begin to grow, and eventually form comparatively large objects, one-sixteenth of an inch in length, just visible to the naked eye. Arrived at this stage they burst out of the muscles, pass forward into the head of the insect below the cephalic ganglion, and so to the base of the labium, which they enter.

The labium is the fleshy part of the proboscis, which does not penetrate the skin when the mosquito bites; but the worms, when biting is going on, come out between this structure and the labellæ, and so wriggle into the wound made by the stylets, the other part of the proboscis. Once under the skin, passage to the lymphatics is easy; and then further growth and differentiation into males and females takes place, with the birth of embryos, which again in turn appear in the peripheral blood in due course, and so complete the cycle. Here again, if you break the chain by destroying the mosquito or intermediate host, the disease must come to an end.

### Tick, Fever, and Relapsing Fever.

Let us now conclude by a word or two on the spirochætes and the pyrosomata, other parasites spread by insects, in both cases by ticks. You all know the spirochætes. They used to be called spirilla; and syphilis, you will remember, is due to one, the *Treponema pallida*. Two of these, the spirochæte of relapsing fever and the spirochæte of African tick fever, merit attention.

It was discovered in South America that fowls may harbour a spirochæte; and it was also shown that this parasite is carried by a tick, passed on to its ova, and from the latter to the next generation of ticks. This was a very important discovery, and it became fairly obvious that the tick fever of Africa might also be due to a spirochæte. This was looked for and was ultimately found. The tick that spreads this is the *Ornithodorus moubata*, a bug-like creature which inhabits the cracks and corners of mud huts and houses. It sucks the blood of infected cases, and just as in the case of the fowl tick (*Argas persicus*), the spirochætes pass to the ova, and so into the young brood of ticks, which therefore in turn become infected, and so can transmit the disease. If one makes a habit of sleeping in native huts in Central Africa, sooner or later some of these infected ticks will bite you and so give this disease.

### Relapsing Fever.

Now what about the ordinary relapsing fever of Europe? This is not spread by ticks, but there is much evidence to show that it is spread by lice. The last case of relapsing fever I saw was a case which had originated in a boarding-house at Greenwich.

The man was a sailor, and in the boarding-house in question there had been a case of relapsing fever from abroad. No doubt lice were present in abundance, and, getting the infection from the imported case, soon transmitted it to the healthy individual. At present we are not certain whether spirochætes are bacteria or protozoa; but they are certainly a class of organism by themselves, and the spread of some of them by insects is of immense interest.

### Cattle Diseases.

As regards the pyrosomata, parasites akin to malaria, but so far never found in man, only in animals, much could be said. They are parasites which cause an enormous mortality in cattle, horses, and other animals in different parts of the world—for example, the *Pyrosoma bigeminum*, the parasite of red-water fever of cattle; the *Pyrosoma parvum*, the cause of East Coast fever in Africa; and the *Pyrosoma canis*, the cause of malignant jaundice in dogs. Before the discovery of the development of malaria in the mosquito, Smith and Kilborne in America had worked out the life-history of the *Pyrosoma bigeminum* completely; and there is no doubt that their discovery led the way to the subsequent one of malaria and the mosquito.

These observers found the parasite, the *Pyrosoma*, in the blood of the infected animals, and showed that ticks which sucked the blood of such animals became infected, the infection passing through the ova to the larvæ and nymphs, and so back to fresh animals. Susceptible cattle taken into an area where this disease exists will soon be bitten by the infected young ticks, and so get the disease and die. The loss to stock-owners and others by this disease alone is enormous, and its presence in countries may often spell financial ruin.

These instances, then, will help you to appreciate what an enormous part is played by insects in the carrying and transmission of some of the most important scourges of the tropics. One point, however, that may be said in favour of insect-borne diseases is that they can be much more easily stamped out than those which are spread in other ways. The cutting of the chain, by removing the insect, is alone sufficient to make the disease disappear.

